# The Epigenetic Role of MiRNAs in Endocrine Crosstalk Between the Cardiovascular System and Adipose Tissue: A Bidirectional View

**DOI:** 10.3389/fcell.2022.910884

**Published:** 2022-07-04

**Authors:** Ursula Paula Reno Soci, Bruno Raphael Ribeiro Cavalcante, Alex Cleber Improta-Caria, Leonardo Roever

**Affiliations:** ^1^ Biodynamics of the Human Body Movement Department, School of Physical Education and Sports, São Paulo University–USP, São Paulo, Brazil; ^2^ Gonçalo Moniz Institute, Oswaldo Cruz Foundation (IGM-FIOCRUZ/BA), Salvador, Brazil; ^3^ Department of Pathology, Faculty of Medicine, Federal University of Bahia, Salvador, Brazil; ^4^ Post-Graduate Program in Medicine and Health, Faculty of Medicine, Federal University of Bahia, Salvador, Brazil; ^5^ Physical Education Department, Salvador University (UNIFACS), Salvador, Brazil; ^6^ Department of Clinical Research, Federal University of Uberlândia, Uberlândia, Brazil; ^7^ Faculty of Medicine, Sao Paulo University, Sao Paulo, Brazil

**Keywords:** microRNA, adipose tissue, obesity, heart, cardiovascular disease, crosstalk, metabolism

## Abstract

Overweight and obesity (OBT) is a serious health condition worldwide, and one of the major risk factors for cardiovascular disease (CVD), the main reason for morbidity and mortality worldwide. OBT is the proportional increase of Adipose Tissue (AT) compared with other tissue and fluids, associated with pathological changes in metabolism, hemodynamic overload, cytokine secretion, systemic inflammatory profile, and cardiac metabolism. In turn, AT is heterogeneous in location, and displays secretory capacity, lipolytic activation, insulin sensitivity, and metabolic status, performing anatomic, metabolic, and endocrine functions. Evidence has emerged on the bidirectional crosstalk exerted by miRNAs as regulators between the heart and AT on metabolism and health conditions. Here, we discuss the bidirectional endocrine role of miRNAs between heart and AT, rescuing extracellular vesicles’ (EVs) role in cell-to-cell communication, and the most recent results that show the potential of common therapeutic targets through the elucidation of parallel and ⁄or common epigenetic mechanisms.

## Introduction

MicroRNAs (miRNAs) are ∼22-nt RNAs that posttranscriptionally repress translation of mRNA targets in eukaryotic and prokaryotic lineages, and are transcripts within longer stem-loop RNA. The latest release of miRBase v22 (https://www.mirbase.org/) contains miRNA sequences from 271 organisms: 38,589 hairpin precursors and 48,860 mature miRNAs. As an example, the human genome contains 1,917 annotated hairpin precursors, and 2,654 mature sequences (Bartel, 2018; Kozomara et al., 2019). The miRNAs are highly conserved between species, preferentially conserving interactions with most human mRNAs, regulating a plethora of developmental processes and health states at molecular, cellular, tissue, and physiological levels (Bartel, 2018). Several miRNAs have a role in cardiovascular biology related to disease etiology and progression, revealing potential as cardiovascular disease (CVD) biomarkers and therapeutic targets (Small and Olson, 2011).

Overweight and obesity (OBT) is a serious health condition worldwide, affecting respectively 1.9 billion and 650 million people, and consequently requiring treatment for several secondary diseases, like CVD, currently the main reason for morbidity and mortality worldwide, which OBT is one of the major risk factors (Hashim, 2017). OBT consists of a body mass index (BMI) > 30 kg/m^2^ associated with a proportional increase of adipose tissue (AT) compared with other tissue and fluids. This condition induces pathological changes in metabolism, hemodynamic overload, cytokine secretion, systemic inflammatory profile, and cardiac metabolism. The AT depots are heterogeneous and they differ in location, secretory capacity, lipolytic activation, insulin sensitivity, and metabolic status, performing anatomic, metabolic, and endocrine functions. (Christensen et al., 2020; Zhang et al., 2020).

Recently, a body of evidence postulated that circulating miRNAs act as endocrine factors, performing endocrine and paracrine crosstalk between cells and tissues. Several circulating miRNAs are implicated in physiological and pathological processes related to metabolism (Ji and Guo, 2019). The concept of the heart as an endocrine organ arises from the discovery of the atrial cardiomyocytes expressing polypeptides with natriuretic properties: ANF and BNP, which at present are biomarkers of cardiac stress (Garciá-Arias et al., 2020). Currently, heart-enriched miRNAs are investigated as biomarkers of cardiac diseases and cardiovascular system (CVS) function regulators and some are known as systemic metabolism regulators (van Rooij et al., 2007; Callis et al., 2009; Grueter et al., 2012).

In this review, we discuss the role of miRNAs in the bidirectional endocrine relationship between heart tissue and AT in circulation, within extracellular vesicles (EVs) or not. On the one hand, we summarized some miRNAs already known to be enriched in AT and their regulatory mechanism on cardiac function and morphology. Furthermore, we discuss the epigenetic regulation performed by cardiac miRNAs in crosstalk with AT, showing the latest evidence about common regulation, parallel mechanisms, and the predictive and therapeutic clinical potential of these tiny and powerful molecules.

### The Relationship Between Obesity, Cardiovascular Diseases, and MiRNAs

OBT is a multi-causal metabolic disease that is associated with hypertrophy and hyperplasia of white AT (WAT) (Rosen & Spiegelman, 2014). Both AT hypertrophy process and hyperplasia occur mainly due to excess food consumption (calorie consumption) and low caloric expenditure (sedentary lifestyle) (Heindel & Blumberg, 2019). These two processes in the AT promote an increase in the number of immune cells in this tissue, inducing a large production of pro-inflammatory cytokines that are released into the circulation (Han et al., 2020, Maurizi et al., 2018).

In addition to the inflammatory process, obesity configures an inadequate supply of oxygen in the AT, inducing hypoxia in these cells, activating hypoxia-inducible factor 1/(HIF-1), which leads to apoptosis of adipose cells and also attenuates preadipocyte differentiation, favoring the increase of the fibrotic process (Buechler et al., 2015).

The association between inflammation and fibrosis leads to AT dysfunction, insulin resistance, and endothelial dysfunction. This scenario paves the way for the development of cardiovascular and metabolic diseases, like hypertension (Brandes, 2014; Longo et al., 2019; Battineni et al., 2021), type 2 diabetes (Avogaro, 2006), and coronary artery disease (CAD) (Ganz & Hsue, 2013). The increase in AT cells also induces hemodynamic overload, due to the increase of systolic volume of the left ventricle (LV), a condition that in the long term will promote pathological cardiac hypertrophy (CH), and systolic and diastolic dysfunction that may go along with heart failure, which in obese individuals is called OBT cardiomyopathy (Ren et al., 2021).

All these pathological processes in OBT are linked to the deregulation of signaling pathways which activates transcription factors, regulates gene expression, and induces pathological profiles of miRNAs. Several miRNAs have already been described as deregulated in AT, in the differentiation of mesenchymal stem cells to preadipocytes (Improta Caria et al., 2018). Several dysregulated miRNAs in OBT were also associated with the inflammatory (Arner & Kulyté, 2015) and fibrotic processes (Caus et al., 2021). Some of these miRNAs have a common expression pattern with other diseases, such as systemic arterial hypertension (Improta-Caria et al., 2021) and type 2 diabetes (Improta-Caria et al., 2022).

### MicroRNAs: Brief History, Biogenesis and Function

MiRNAs were initially discovered during analyzes of the progression from the first larval stage (L1) to L2 of the nematode *Caenorhabditis elegans,* in which the decrease in the expression of the LIN-14 protein was essential for the development of the worms. In addition, the downregulation of LIN-14 occurred due to the progressive transcription of another gene, known as *LIN-4,* short single strand RNAs, which were not translated into protein and adversely transcribed two small RNAs about 22–61 nucleotides in length, and with complementarity in the 3′-untranslated regions (UTR) of the LIN-14 mRNA (Lee et al., 1993). Subsequently, the hybridization in these complementary regions was linked with a decrease in the LIN-14 protein content, without impacting the decrease in the expression of its transcript (Wightman et al., 1993).

Following these findings, other researchers identified another small RNA known as let-7 that promoted the adult larval stage of *C. elegans* (Reinhart et al., 2000). Interestingly, let-7 has also been identified in humans (Pasquinelli et al., 2000), drawing the attention of several researchers around the world. In the following years, several research groups began to further investigate the role of these small RNAs in different organisms, demonstrating their biogenesis and their regulatory function (Lee et al., 2002; Ambros, 2004; Pfeffer et al., 2005; Roush & Slack., 2008; Morlando et al., 2008).

MiRNA biogenesis is a multi-molecular-step process that starts in the nucleus and ends in the cytoplasm with the synthesis of mature miRNA (Kim, 2005). The miRNAs are processed from a precursor molecule, referred to as primary transcript (pri-miRNA), which is transcribed initially by the RNA polymerase II (Lee et al., 2004; Treiber et al., 2019). This enzyme transcribes the pri-miRNA, which contains one or more sequences that are enveloped in a stem-loop structure. In the nucleus, pri-miRNA receives two cleavages between the lower and upper stems of its structure by Drosha, an RNase III-like enzyme (Davis et al., 2010). It acts together with cofactors including an essential subunit protein, the DiGeorge syndrome chromosomal region 8 (DGCR8). Drosha and DGCR8 processing steps form the microprocessor complex to mature the pri-miRNA into pre-miRNA (Denli et al., 2004; Wang et al., 2007). After processing by Drosha, a long transcript is enveloped in a stem-loop intermediate structure, an ∼75 nucleotides called precursor (pre-miRNA) (Krol et al., 2010).

The product of Drosha cleavage is exported to the cytoplasm by Exportin 5, where the next cleavage occurs by Dicer, an RNase III-like endonuclease (Ketting et al., 2001; Yi et al., 2003; Zeng and Cullen, 2004; Okada et al., 2009; Fisher et al., 2011). Dicer cleaves the pre-miRNA hairpin into a miRNA duplex about 25 nucleotides in length (Hutvágner et al., 2001). After the Dicer process, the mature miRNA is incorporated into the RNA-induced silencing complex (RISC), generating the (mi-RISC) complex. This mi-RISC induces downregulation of target genes, modulating gene expression. (Chakravarthy et al., 2010; Horman et al., 2013). Next, the mature miRNA associated with miRISC binds to the 3′-UTR of the target mRNA causing degradation, deadenylation, or inhibition of translation of this gene. Impressively, a single miRNA can have multiple mRNA targets, inducing epigenetic regulation of gene expression at the post-transcriptional level and modulation of several signaling pathways (Samanta et al., 2016; Bartel, 2018).

### Adipose Tissue Diversity of Depots and Function

AT is a crucial organ in human anatomy as it plays a key role in regulating body energy and glucose homeostasis. It has effects on physiology and pathophysiology by displaying relevant tasks in lipid handling, energy storage compartment, insulation barrier, and secretion of endocrine mediators such as adipokines or lipokines (Vegiopoulos et al., 2017). Finally, AT is considered a highly active metabolic and endocrine organ (Kershaw and Flier, 2004).

AT is composed of several cells and components, including adipocytes (the most common cell type), lymphocytes, macrophages, fibroblasts, endothelial cells, and extracellular matrix (Corrêa et al., 2019). Morphologically, some types of AT have been identified in humans, namely white, brown, and beige or “brite” (brown-in-white). This classification is based on the colorful diversity and predominant presence of adipocytes: in WAT, there is a significant presence of white adipocytes; in brown AT (BAT), brown adipocytes are mainly present. Considering the plasticity of AT and its ability to proliferate, differentiate, and transdifferentiate, the third type of adipocyte, beige (BeAT) results from white adipocytes that have acquired phenotypic brown features in response to different *stimuli*, in a process called “browning” (Pilkington et al., 2021).

The WAT can also be classified by location*,* as subcutaneous (under the skin) and visceral/omental (intra-abdominally, adjacent to internal organs). In addition, WAT is confined to defined depots in healthy individuals but in certain conditions like OBT and lipodystrophy, WAT mass can ectopically increase in areas such as the visceral cavity, including intrahepatic fat, epicardial fat (EAT) in the pericardium, perivascular fat (PVAT) surrounding major blood vessels, and visceral fat (VAT), which comprises mesenteric fat, omental fat, and retroperitoneal fat (Chait and den Hartigh, 2020).

AT types can be found in specific anatomical sites throughout the body and each one has displayed distinct characteristics and functions: whereas WAT adipocytes are associated with storage and release of energy during fasting periods (Torres et al., 2015), BAT adipocytes have thermogenic properties, burning glucose and lipids to maintain thermal homeostasis during periods of low temperature and hibernation (Rosen and Spiegelman, 2014). Despite similarities to brown adipocytes, BeAT adipocytes can undergo a thermogenic or storage phenotype depending on environmental conditions (Zoico et al., 2019).

As an endocrine organ, AT responds to physiological cues or metabolic stress, releasing endocrine factors that regulate energy expenditure, appetite control, glucose homeostasis, insulin sensitivity, inflammation, and tissue repair. WAT and thermogenic BAT and BeAT also secrete endocrine molecules, such as adipokines, lipokines, miRNAs, and other noncoding RNAs.

Recent findings emphasize the endocrine role of white versus thermogenic adipocytes in conditions of cardiac health and disease (Scheja and Heeren, 2019). Furthermore, AT secretes molecules, directly or *via* extracellular vesicles (EVs) (including exosomes and nano-sized vesicles generated from late endosomes), containing proteins, lipids, and nucleic acids, such as miRNAs which recently have been investigated as epigenetic mediators of endocrine and paracrine effect between AT and other tissues, like the cardiac (Hartwig et al., 2019; Zhang Y. et al., 2019).

### Exosomes and Circulating MiRNAs as Epigenetic Mediators in the Cardiovascular System and Adipose Tissue Crosstalk

The last decade increased understanding of the adipocytes’ role in health and disease. There is growing evidence implicating extracellular vesicles miRNAs (EVs-miRNAs) and circulating miRNAs mediating intercellular and inter-organ communication. These miRNAs are classified as extracellular miRNAs, since they are detected in an extracellular environment, as biological fluids and cell culture media.

EVs are systemic messengers that can deliver signaling molecules. Exosomes, microvesicles, and apoptotic bodies are the most important EVs and have distinct biogenesis pathways sizes and types (Mathieu et al., 2019). Adipocytes are a major source of EVs-containing miRNA in circulation. An increasing number of studies have shown that EVs and their cargo play important roles in cellular crosstalk between cells and tissues, and therefore can regulate disease and health conditions. Nevertheless, the detailed mechanisms in these complex fields are far from being completely elucidated, comprising the interaction between the biogenesis of miRNAs and the biogenesis and maturation of EVs in several tissues and cells related to CVD and AT (Turchinovich et al., 2012; Kranendonk et al., 2014; Fang et al., 2016; Sluijter et al., 2018).

The accurate characterization of EVs is limited by the technical difficulty in isolating and characterizing pure tissue-specific populations and their subtypes since the current methods are mainly based on the co-isolation of EVs of distinct subcellular origins. Many studies use ‘exosome’ referring to a mixture of small EVs of both exosomal and nonexosomal nature, due to poor specificity of the physical processes for isolation and purification of EVs. Thus, unless the EVs’ origin has been clearly stated, it may be preferable to use the generic term ‘small EVs’ instead of ‘exosomes’, which range 10–200 nm in diameter (Mathieu et al., 2019). Given the complexity of processes and technical limitations to investigating exosomal miRNAs, it is a promising and challenging field to elucidate tissue crosstalk, including AT and CVD. Furthermore, there is a recent and growing body of evidence that miRNAs content in AT exosomes plays key roles in cardiovascular processes, clinically reinforcing obesity as a CVD risk factor. Similarly, there are established cardiac-enriched miRNAs that can regulate AT depots *via* systemic metabolism and other biological processes, which will be addressed in the last topic.

### The Endocrine Function of Adipose Tissue-Enriched MiRNAs on the Cardiovascular System

In OBT, AT increases the size and number of adipocytes, storing more triglycerides. Additionally, AT synthesizes and releases hormones called “adipokines”, like leptin and adiponectin, and other factors which affect biological pathways, at autocrine, paracrine, and endocrine levels, including the regulation of whole-body energy homeostasis (Patel et al., 2017). In this condition, AT can become unhealthy as adipocytes lose their ability to store triglycerides adequately, have impaired energy expenditure, and become insulin resistant. Consequently, fatty acids are released into the circulation and accumulate in other organs, causing cellular stress, disturbed metabolism, and altered secretion of endocrine factors, regarded as a hallmark of chronic metabolic and CVD (Reilly, 2017; Scheja and Heeren, 2019).

The evidence postulates that AT-derived circulating miRNAs are currently described as a new form of adipokines (Thomou et al., 2017). Circulating (or extracellular) miRNAs are freely and/or carried within exosomes, lipoproteins, and blood cells, from cells that express them to cells that receive them (Sohel, 2016). Recent evidence shows that AT is the main source of all circulating exosomal miRNAs, in humans and mice. Knockout Dicer-deficient (ADicerKO) mice present lipodystrophic phenotype and AT-deficient miRNA processing, decreasing AT-derived miRNA expression. Similarly, lipodystrophy decreases the levels of circulating exosomal miRNAs compared to healthy people. Among 653 miRNAs in serum EVs, 419 decreased in fat-specific DicerKO mice, 88% by more than four-fold. (Thomou et al., 2017). In summary, 216 miRNAs decreased in patients with lipodystrophy compared to healthy people and 30 common miRNAs decreased between ADicerKO and patients, which shows that AT releases numerous miRNAs *via* exosomes that may be involved in cell-to-cell epigenetic regulation and the regulation between health and CVD (Thomou et al., 2017). Inversely, transplantation of wild-type mice-derived WAT and BAT into ADicerKO mice restored exosomal miRNAs and improved glucose tolerance, showing evidence that AT-miRNAs are also crucial for regulating energy metabolism, and their expression is associated with a proper function of AT (Thomou et al., 2017). The transplantation with BAT and WAT into ADicerKO mice restored miR-325 and miR-743b (predicted to target UCP-1) and miR-98 (predicted to target PGC1α) for BAT and miR-99 for BAT and WAT, suggesting that AT-secreted miRNAs may have both paracrine and endocrine actions. In addition, pediatric obesity presents an increase in 16 circulating miRNAs previously associated with nonalcoholic fatty liver disease, reinforcing that free and exosomal miRNAs are released from AT cells to influence several tissues and biological processes, including cardiovascular health regulation (Thompson et al., 2017).

MEG-3 is a long-noncoding RNA involved in the imprinting of maternal genes that sponges miR-325. Hypoxia-reperfusion in H9c2 cardiomyoblast cells increases MEG-3, decreases miR-325, and increases the protein content of target TRPV4. TRPV4 is a Calmodulin-dependent Ca^2+^ channel that regulates Ca^2+^ concentration in excitable cells and, concomitantly in adipocytes, regulates the expression of chemokines and cytokines related to pro-inflammatory pathways. This entire process denotes not only miRNAs crosstalking but also that it could protect or enhance the response to ischemic injury (Zhou et al., 2021). Furthermore, Hsa-miR-325 is elevated in normal pregnancies and decreases in preeclampsia patients, being implicated in preeclampsia etiology (Lázár et al., 2012). In ApoE^−/−^ mouse with atherosclerosis, miR-325 increases in arterial tissues of atherosclerotic mice, and miR-325 inhibition reduces the contents of total cholesterol, triglyceride, low-density lipoprotein, and CRP, IL-6, IL-1β and TNF-ɑ levels in mouse serum. *In vitro* miR-325 inhibition decreased the lipid content in RAW264.7 macrophage cells *via* KDM1A to reduce SREBF1 expression and activated the PPARγ-LXR-ABCA1 pathway. KDM is a demethylase that regulates lipogenic genes (Pu et al., 2021). LXRs are expressed in the murine heart in the basal state and are activated by myocardial infarction, also associated with an intracardiac accumulation of lipid droplets and protection against myocardial ischemia-reperfusion injury (Lei et al., 2013). PPARγ is a nuclear receptor that stimulates lipid and glucose utilization by increasing mitochondrial function and fatty acid desaturation pathways, being crucial for cardiac function and metabolism (Montaigne et al., 2012). PPARγ also is a regulator of AT signaling and plays a crucial role in insulin sensitivity, making it an important therapeutic target. Moreover, PPARγ activation increases cardiac hypertrophy and oxidative stress in mice. Cocultures of adipocytes and cardiomyocytes showed that stimulation of PPARγ signaling in adipocytes increased miR-200a expression and secretion. Delivery of miR-200a in adipocyte-derived exosomes to cardiomyocytes inhibits TSC1 and activates the mTOR pathway, leading to CH. Inhibition of miR-200a abrogated the CH, clarifying that the miRNA cargo in EVs can change cardiac phenotypes and showing evidence of endocrine crosstalk between heart and AT performed by EVs (Fang et al., 2016).

In a single study using a rat model in a time course in transverse constriction of the Aorta, cardiac miR-743b acutely increased over 2-fold after 5 days compared with 10, 15, and 20 days of pressure overload. The increase was associated with pathological remodeling and CH; however, additional investigation is needed to assess if EVs circulating AT miR-743b has some additive effect on the cardiac remodeling phenotype (Feng et al., 2014).

In a murine model for cardiac allograft transplantation, miR-98 plays a role in regulating interleukin (IL)-10 expression in B cells (B10 cell) after heart transplantation. The miR-98 inhibition, cortisol inhibition, and transfer with B10 cells enhanced the survival rate and time of transplanted mice (Song et al., 2017). In the first atlas of miRNA profile using internal mammary artery from 192 CAD disease patients, miR-98 was significantly correlated with acute myocardial infarction occurrence, suggesting that this AT-enriched miRNA is also related to the regulation of cardiac function (Neiburga et al., 2021). In addition, miR-98 in human fibroblasts inhibits TGF-β1-induced differentiation and collagen production of cardiac fibroblasts targeting TGFβR1, performing a role in the fibrotic phenotype, present in all cardiac diseases (Cheng et al., 2017). Finally, miR-98 is downregulated in myocardial infarct injury (MII) and neonate primary culture of cardiomyocytes in response to H_2_O_2_ stress. Additionally, miR-98 overexpression protected cardiomyocytes against apoptosis by its target Fas, inhibiting the Caspase-3 apoptotic pathway (Sun et al., 2017).

Adipocytes-enriched miRNAs play an essential role in regulating gene expression and cell-to-cell communication, through mRNA downregulation, therefore interfering in a multitude of biological processes (Eichhorn et al., 2014; Heyn et al., 2020). OBT changes drastically the profile of the AT-enriched miRNAs, influencing circulating and exosomal miRNAs content. Consequently, aberrant intra- and extracellular miRNAs profiles can induce crosstalk between AT, liver, skeletal muscle, and other organs, which impacts the development of different cancers and metabolic CVD (Muralimanoharan et al., 2015; Sala et al., 2021). There is evidence that OBT and weight loss alter the profile of circulating miRNAs in humans and mice, affecting pathways associated with body mass index (BMI), and others such as percent fat mass, waist-to-height ratio, and plasma adipokine levels. The compared whole profile of circulating miRNAs pre- and post-surgery weight loss in 6 morbidly obese patients showed that the most relevant circulating miRNAs differences were the increased expression of miR-142-3p, miR-140-5p, and miR-222 and the decreased circulating concentrations of miR-221, miR-15a, miR-520c-3p, miR-423-5p, and miR-130b (Ortega et al., 2013; Lörchner et al., 2021). Additionally, the plasma concentrations of all were associated with BMI and most of them with fat mass and waist circumference. Interestingly, the 2 major targets for the *in silico* intersection between miR-142-3p and miR-140-5p (LIFR) and between miR-15a and miR-520c-3p (VEGFA) were significantly associated with the circulating values of their specific transcriptional regulators. The plasma content of LIFR (a cardioprotective IL-6 receptor), was negatively correlated with the circulating concentrations of miR-142-3p, and miR-140-5p, whereas miR-15a and miR-520c-3p were negatively correlated to circulating VEGFA (Ortega et al., 2013; Lörchner et al., 2021). There are several AT-enriched and OBT-related miRNAs with concomitant roles in heart phenotypes. The large-scale mapping of the epigenetic regulations between heart and AT at the systemic level may shed light on corrective post-translational multi-gene therapies.

Another study elucidates the metabolic influence in endocrine crosstalk of miRNAs performed between AT and CVS and delineates a molecular mechanism by which dysfunctional adipocytes could exacerbate myocardial infarct injury (MII) *via* EVs-miRNAs. The transplantation of diabetic epididymal fat or intramyocardial or systemic administration of diabetic adipocyte EVs in MII mice exacerbated the injury in nondiabetic mice. Inversely, the injection of an EVs’ biogenesis inhibitor abrogated the additional deleterious effect and improved cardiac function post-MI, increasing dP/dt (max) compared with MII vehicle mice. MiR-130b-3p was implicated in the mechanism due to an increase in diabetic patients’ plasma and mice diabetic adipocyte, serum, and EVs. In addition, mimic for miR-130b-3p increased and miR-130b-3p inhibitor decreased MII injury, *via* direct targets such as AMPKα1/α2, BIRC6, and UCP3, showing a direct mechanistic relationship between miRNAs, AT, and cardiac injury (Gan et al., 2020).

Considering the established EAT and PAT bidirectional effects on cardiovascular health *via* the production and secretion of adipokines (Patel et al., 2017), and AT circulating miRNAs emerging as multilevel epigenetic regulators with functional and structural roles in CVS, additional investigation into the miRNAs crosstalk between AT and CV tissue is crucial and has clear clinical potential as therapeutic targets and biomarkers for the assessment of metabolic disorders and obesity-related diseases. In this way, the next topics will show and discuss the bidirectional relationship between AT and CVS miRNAs, rescuing the functional evidence on this issue in an extracellular environment.

### Epicardial and Pericardial Adipose Tissue MiRNAs

Epicardial and Pericardial AT (EAT and PAT, respectively) are anatomically and biochemically distinct and have different cellular origins. EAT lies between the outer wall of the myocardium and the visceral layer of the pericardium, while PAT lies between the visceral and parietal pericardium. Since no fascia separates the tissues, EAT is in direct contact and communication with the myocardium, in the atrioventricular and interventricular grooves, and alongside the coronary arteries of the human heart. PAT splits to form the parietal pericardium and the outer thoracic wall. EAT differentiates from splanchnopleuric mesoderm, whereas PAT arises from the primitive thoracic mesenchyme (Iacobellis, 2009; Zhang et al., 2020).

Translational studies are also interesting approaches to overcome challenges and current limitations to evidence EVs’ crosstalk between AT and CVS among species. Microscopic analyses show inflammatory, fibrotic, and apoptotic phenotypes in fresh and cultured EAT tissues from CVD and Atrial Fibrillation (AF) patients. AF-EVs presented a high expression of profibrotic (miR-146b) and low expression of antifibrotic miRNAs respectively (miR-133a, miR-29a). Concomitantly, EVs harvested from AF-EAT patients exacerbated fibrotic phenotype in rats and changed electrophysiological properties facilitating arrhythmias in cardiomyocyte-hiPSC culture, reinforcing the evidence of the paracrine and endocrine effect of AT miRNAs in cardiac cells predisposing to the disease, i.e., showing the crosstalk between EAT and heart phenotypes *via* miRNAs as endocrine effectors (Shaihov-Teper et al., 2021). The role of miR-29a, -133a and -133b, and -146 on cardiac fibrosis, function, and remodeling is well established and does not require additional comments (Carè et al., 2007; Van Rooij et al., 2008; Li et al., 2012; Feng et al., 2017).

In humans, the evidence that the EAT is an active endocrine organ is robust. EAT is metabolically active and a source of several adipokines, potential interactions through paracrine or autocrine mechanisms between epicardial fat and the myocardium regulating between healthy and disease state. The PAT as a source of adipokines is still partially unknown, being more related to atherosclerosis and CAD. However, it is possible that PAT interacts paracrinally with the pericardium tissue and EAT. EAT is very metabolically active, therefore, lipolysis and fatty acid synthesis are greater in EAT compared to visceral fat, and PAT, and EAT adipocytes are smaller than other AT cells (Christensen et al., 2020).

Considering that PAT has more potential to release inflammatory cytokines than subcutaneous fat, it is interesting to investigate its interaction with EAT to explain gene etiology and CAD regulation (Ding et al., 2008; Iacobellis, 2009; Hassan et al., 2020; Zhang et al., 2020). An increased EAT thickness has become a new risk factor for CAD. A study already aimed at identifying the miRNA profile role of EAT dysfunction as a CAD marker. EAT miRNA array profiles from 150 CAD sudden cardiac death victims and 84 non-CAD-sudden death controls were prospectively enrolled at autopsy and showed the following EAT miRNA profile candidates for dysregulation: miR-34a-3p, miR-34a-5p, miR-124-3p, miR-125a-5p, miR-628-5p, miR-1303, miR-4286 related to atherosclerosis and plaque destabilization pathways. MiR-34a-3p and miR-34a-5p were higher in CAD, were positively correlated with age, and were validated as biomarkers of CAD, independently of thickness and plaque formation (Marí-Alexandre et al., 2019).

MiR-34a is regarded as an effector for endocrine AT-CVS crosstalk. The evidence shows it as reinforcing loss of function in CVS by several pathways. MiR-34 levels are relatively low in the CVS, but recently they have been reported to be increased in cardiovascular disorders. MiR-34a is a predictive biomarker in mice after myocardial infarct injury (MII) and presents low expression in healthy hearts (Li et al., 2015; Qipshidze Kelm et al., 2018). The inhibition miR-34 family has been investigated as therapeutic for CVD by regulating apoptosis, telomere waste, DNA damage (targeting PNUTS), inflammatory response (KLF4, SEMA4b, BCL6), inotropic and excitability (Vinculin), and cardiac fibrosis (ALDH2) (Bernardo et al., 2012; Boon et al., 2013; Li et al., 2015; Qipshidze Kelm et al., 2018). In mice, they have the same seed sequence, suggesting their common target mRNAs. In human beings, miR-34a and miR-34c have the same seed sequence, and miR-34b has three short nucleotide sequences identical to miR-34a and miR-34c, showing that the target mRNAs may change between species and miRNAs (Li et al., 2015). The circulating miR-34a expression in AT progressively enhances with the development of diet-induced OBT. Inversely, adipocyte-specific miR-34a-KO mice are resistant to OBT-induced glucose intolerance, insulin resistance, and systemic inflammation, related to a significant shift in the polarization of adipose-resident macrophages from pro-inflammatory M1 to anti-inflammatory M2 phenotype (Pan et al., 2019). Finally, miR-34a can inhibit fat browning by suppressing the browning activators FGF2 and SIRT1 in mice, showing a dual role as a therapeutic target for CVD and OBT (Fu et al., 2014).

MiR-99 family comprises miR-99a, miR-99b and miR-100. They show very similar sequences and identical seeds. MiR-100 has one different nucleotide compared to miR-99a, and four compared to miR-99b. MiR-99a, in turn, differs from four nucleotides compared to miR-99b. This family, in addition to being enriched in AT, also concomitantly shows a regulatory role between physiological and pathological CH, with apoptosis and growth processes in both *in vitro* and *in vivo* settings. (Ramasamy et al., 2015, 2018). Swimming exercise training showed a miRNA profile by RNAseq in which miR-99b and miR-100 were downregulated (Ramasamy et al., 2015). In addition, physiological and pathological CH was induced in H9c2 cells by treatment with α2-macroglobulin and Isoproterenol, respectively. The miR-99b and miR-100 were downregulated in physiological CH and upregulated in pathological CH targeting AKT-1. Upstream, EGR-1 superexpression binds to the promoter and induces miR-99b and miR-100 expression, and downstream, AKT-1 silencing replicates the effect of overexpression of miR-99, showing the mechanism by which this regulation occurs through this AT-enriched family of miRNAs (Ramasamy et al., 2018).

Therefore, a clipping was performed here to demonstrate the potentiality of the crosstalk between AT and heart *via* miRNAs. There is a vast field to be clarified in this sense, with very comprehensive clinical perspectives regarding therapies and detection methods. In the next topic, we will discuss the other side of bidirectional crosstalk from the heart to AT.

### Cardiac Enriched MicroRNAs: The Heart as an Endocrine Organ

The evidence of the heart as an endocrine organ emerged from studies that showed that the atrial cardiomyocytes in the mammalian heart could perform roles similar to endocrine cells, by the expression of ANF, BNP, and CNP in circulation. These molecules, known as natriuretic peptides, displayed paracrine functions related to blood volume regulation, cardiac output, and serum concentrations of sodium and total body water. These studies were the guideline for identifying new molecules linked to the contractile function of the heart (Garciá-Arias et al., 2020).

The first evidence of a miRNA as a regulator of systemic metabolism related to the endocrine role of the heart emerged by miR-208a, which pharmacologic inhibition by injections induced resistance to obesity in animals fed with a high-fat diet (Grueter et al., 2012). MiR-208a is cardiac-specific and is encoded by the α-myosin heavy chain (MHC) gene. This miRNA up-regulates β-MHC by directly targeting PURβ and SOX-6 together with miR-208b and miR-499, also called myomiRs, which share a similar seed sequence. β-MHC has an ATPase activity slower than α-MHC and is a pathological CH and cardiac stress marker (Van Rooij et al., 2007; van Rooij et al., 2009). Therefore, miR-208a is considered an epigenetic biomarker of myocardial stress, having a high predictive potential in several pathological conditions (Callis et al., 2009; Ji et al., 2009; Boštjančič et al., 2010; Satoh et al., 2010). Grueter et al. (2012) by several mechanism studies, including transgenic mice models, showed that MED13 is a target of miR-208, which triggers systemic and cardiac metabolic actions of miR-208a, and indirectly regulates β-MHC expression. In addition, cardiac-specific gain and loss of function of MED13 in mice established a crucial role in the governance of whole metabolism and the control of energy expenditure pathways by regulating the action of nuclear receptors. Cardiomyocyte-specific overexpression of MED13 in mice conferred a lean phenotype by enhancing metabolism in white AT and the liver, and O_2_ consumption, without increasing food consumption. The epigenetic mechanism of systemic metabolism regulation *via* miR-208 is not yet fully elucidated. However, miR-208a increases in several cardiovascular diseases, including heart failure. Considering the systemic, cardiac, and metabolic changes arising from severe cardiac diseases, there is evidence of a systemic-metabolic down-regulation of transcription role performed by nuclear receptors on metabolic genes profile and a possible relationship with mitochondrial dysfunction (Gan et al., 2013). Additionally, there is a glimpse of miR-208a inhibition in a clinical perspective for OBT and CVD by metabolic gene expression, considering that the challenge to therapies towards miRNAs lies in controlling the expression in an acceptable physiological range, beyond improved oligonucleotides, deliveries, and vectors.

Other noncardiac-specific myomiRs that are highly expressed and involved in AT regulation are miR-1, and miR-133, miR-378. In addition, there are others that present lower baseline expression, such as miR-208a, and for which the studies show involvement in metabolism in disease, such as miR-21, and the miR-34 and miR-30 families, which may play a reinforcing role in regulating phenotypes.

MiR-133 was first characterized in mice. Its homologs were identified in several other species, including the human genome in which miR-133 genes comprise miR-133a-1, miR-133a-2, and miR-133b located on chromosomes 18, 20, and 6. Importantly, miR-133a-1 and miR-133a2 have identical nucleotide sequences, whereas miR-133b differs in the last 2 nucleotides at the 3′-terminus. MiR-133a-1, miR-133a-2, and miR-133b are bicistronically transcribed with miR-1-2, miR-1-1, or miR-206, with low genomic distances between the miRNA coding regions. Cardiac miR-133 has a crucial role in cardiac remodeling in response to several stresses (Matkovich et al., 2013). The decreased expression of miR-133 is correlated with the increased severity of HF and a high NT-proBNP concentration (Danowski et al., 2013). In animal models, miR-133 also regulates cardiac fibrosis, electrical activities apoptosis, and gene reprogramming by targeting a plethora of targets (Li et al., 2018). Outside CVS, miR-133 controls BAT fate determination in skeletal muscle satellite cells (SMSC) targeting the *PRDM16* gene, regulating the choice between myogenic and brown adipose determination. Since brown adipocytes derive from myogenic progenitors during embryonic development, *PRDM16*, highly expressed in WAT and myogenic cells, performs the role of a crucial regulator in BAT adipogenesis. Thus, miR-133 also becomes an important therapeutic target to treat obesity, in addition to cardiovascular function (Yin et al., 2013). As the miR-133 family is highly expressed in muscle tissues and decreases both CH and skeletal muscle hypertrophy, and this miRNA regulates the differentiation and proliferation by cell cycle targets and transcription factors, this family is an interesting target for whole approaches for CVD, metabolic disease and obesity.

The miR-378 family also is highly expressed in heart and has 11 members (miR-378a-3p/b/c/d/e/f/g/h/i/j and miR-422a). Although they are encoded by different genomic *loci*, they share identical seed sequences, and the family is conserved between humans and rodents. MiR-378 family targets 4 mRNAs of the MAPK pathway: MAPK1, IGF1, and GRB2 displaying epigenetic regulation of CH in cardiomyocytes. Concomitantly, the metabolic regulation is mediated by PGC1α and KSR1, being ERRα-dependent and MAPK-independent, suggesting that common molecular regulatory points intersect CH and metabolism (Fisher et al., 2011). Plus, the PGC-1β gene encodes miR-378-3p and miR-378-5p, with the latter being responsible for counterbalancing its metabolic actions. Knockout mice for miR-378-3p and miR-378-5p, like miR-208a, are resistant to high-fat diet-induced obesity and exhibit a higher oxidative capacity for fatty acid metabolism in insulin-target tissues. This role seems to be performed by multiple targets, pointing out carnitine O-acetyltransferase (CRAT) and MED13, both increased in the livers of miR-378-3p/378-5p KO mice (Carrer et al., 2012).

MiR-21 is highly expressed in CVS. It is encoded by the VMP1 gene in chromosome 17 and is highly conserved between vertebrates. MiR-21, different from other tissue-specific miRNAs, is expressed in several mammal organ systems: heart, spleen, the small intestine, and colon, and many functional studies have identified miR-21 as an oncomiR. In the CVS, it is associated with the regulation of proliferative vascular disease, atherosclerosis, coronary heart disease, post angioplasty restenosis, and transplantation arteriopathy by targeting PTEN and PDCD4, and CH by targeting SPRY2 (Cheng and Zhang, 2010a). In this regard, some authors already showed that miR-21 inhibition in mouse hearts reduced cardiomyocyte size and the heart weight under CH conditions, and that pathological CH was induced by miR-21 by stimulating MAPK signaling in cardiac fibroblasts (Thum et al., 2008). If, on the one hand, miR-21 inhibition is therapeutic for cardiomyocytes, the same does not happen to adipocytes. An *in vitro* study shows that overexpression of miR-21 in glucose-insulin overloaded cells significantly increased insulin-induced glucose uptake and decreased PTEN protein expression, improving the metabolic phenotype of adipose cells, and the underlying mechanisms of versatile miRNA-21 in both tissues and their communication by circulation need further investigation (Cheng and Zhang, 2010b).

Finally, another cardiac-enriched family also implicated in the regulation of AT is the miR-30. This family is involved in ventricular CH by several mechanisms: autophagy, apoptosis, oxidative stress, and inflammation, associated with ischemic heart disease, hypertension, diabetic cardiomyopathy, and antineoplastic drug cardiotoxicity. The miR-30 family expression decreases in CH and myocardial ischemia/reperfusion, being permissive to a variety of targets to perform roles in the disease and also compensatory effects (Zhang X. et al., 2019). Beyond the role in CVS, the miR-30 family plays a role in AT regulating adipocyte differentiation, since its expression increases in the differentiation of human AT-derived stem cells into adipocytes. The inhibition of miR-30a and miR-30d in human multipotent adipose-derived stem cells reduced lipogenesis, and inversely, the overexpression of miR-30a and miR-30d family members promoted lipogenesis by targeting the transcription factor RUNX2 (Zaragosi et al., 2011). The miR-30b and -30c also increase thermogenic gene expression in primary adipocytes during adipocyte differentiation, cold exposure, or by the β-adrenergic receptor. Furthermore, the knockdown of miR-30 family members (including miR-30b and miR-30c), inhibited the expression of uncoupling protein 1 (UCP1) and cell death-inducing DFFA-like effector a (CIDEA) in brown adipocytes, by directly targeting RIP140, a nuclear receptor that acts as a co-regulator of lipid and glucose metabolism, showing a clear role in regulating BAT function (Hu et al., 2015). In summary, the miR-30 family performs a role in adipogenesis and regulates BAT function, showing that it may be another potential therapeutic target for regulating and clarifying lipid metabolism.

## Conclusion

**FIGURE 1:**
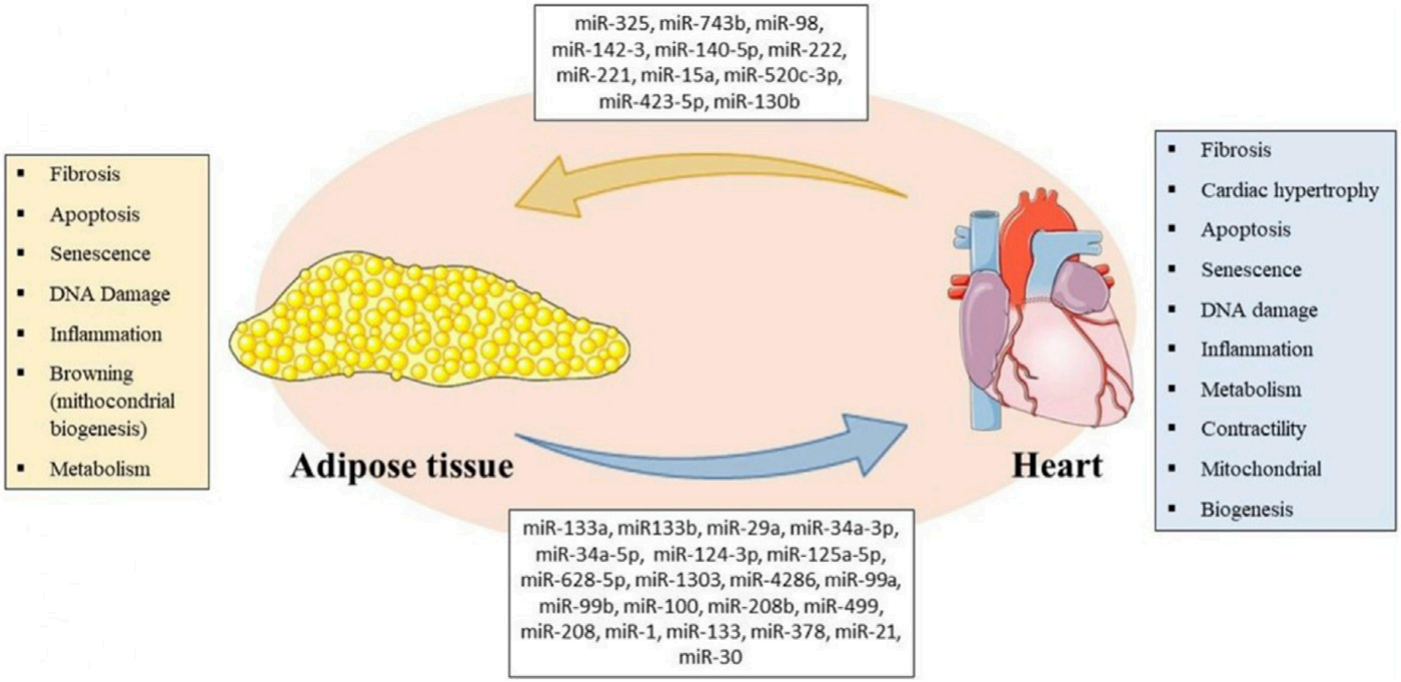
Representative Scheme of miRNAs secreted by AT and heart with their respective biological processes involved in bidirectional crosstalk between tissues. Parts of the figure were drawn using pictures from Servier Medical Art (https://smart.servier.com/). Servier Medical Art by Servier is licensed under a Creative Commons Attribution 3.0 Unported License.

We elucidated some regulatory miRNAs and their endocrine roles by bidirectionally acting on the CV system and AT to regulate metabolism and several biological processes between phenotypes in health and disease (Figure 1; Table 1). The epigenetic relationship between tissues and the whole role performed by miRNAs and other regulatory RNAs remains a very complex field with several gaps to be investigated. It is worth mentioning that this bidirectional relationship is carried out through the circulation, and it is likely that the miRNAs that are part of crosstalk come not only from the AT and the heart and their cells but also from other tissues such as skeletal muscle, liver and the neuroendocrine axis. In addition, free or within EVs, miRNAs are not the only molecules involved in crosstalk, and genes, proteins, and other effector molecules can be carried, such as myokines and adipokines. Thus, the crosstalk is multilevel and involves not only the heart and AT, but is systemic. We discussed an interesting molecular basis that could partially explain the intricate, frequent and worldwide relationship between obesity and CVD. It remains unclear if the cardiac miRNAs are released within EVs, and publications regarding the role of EVs in these miRNAs mechanisms are emerging. These issues are of great interest, both mechanistically in a basic science view as in a clinical perspective, since CVD also may induce metabolic and morphological changes, and inversely, metabolic and morphological changes may induce CVD.

**TABLE 1:** miRNAs secreted by adipose tissue and heart and targets potentially involved in bidirectional crosstalk.

Adipose tissue-enriched miRNAs with cardiovascular functions			
miR	Target	Model	References
miR-325 miR-743b	UCP-1 TRPV4	Mice	Thomou et al. (2017)
		Rat	Feng et al. (2014)
		cells	Cheng et al. (2017)
			Zhou et al. (2021)
miR-98	PGC1α TGFβR1	Mice cells	Cheng et al. (2017)
			Song, et al. (2017)
			Neiburga et al. (2021)
			Sun et al. (2017)
miR-142-3p	LIFR VEGFA	Humans	Lörchner et al. (2021)
miR-140-5p			
miR-222			
miR-221			
miR-15a			Ortega et al. (2013)
miR-520c-3p			
miR-423-5p			
miR-130b			
miR-200a	TSC1	Cells	Fang et al. (2016)
miR-130b-3p	AMPLα1/α2BIRC6 UCP3	Mice	Gan et al. (2013)
Epicardial and Pericardial Adipose Tissue miRNAS			
miR	Target	Model	References
miR-133a		Human explants	Shaihov-Teper et al. (2021)
miR-133b			
miR 29a			
miR-34a-3p	PNUTSKLF4 SEM4b BCL6 ALD2	Humans mice	Mari-Alexandre et al. (2019) Bernardo et al. (2012) Boon et al. (2013) Li et al. (2015) Qipshidze Kelm et al. (2018)
miR-34a-5p			
miR-124-3p			
miR-125a-5p			
miR-658-5p			
miR-1303			
miR-4286			
miR-99a	AKT-1	rat cells	Ramasamy et al., 2015, 2018
miR-99b			
miR-100			
Cardoac-enriched miRNAs			
miR	Target	Model	References
miR-208a	PURβ	Mice	Van Rooij et al. (2007)
miR-208b	SOX-6		Van Rooij et al. (2009)
miR-499	MED13		Grueter et al. (2012)
miR-1	PRCM16	cells	Yin et al. (2013)
miR-133			
miR-378	MAPK1	Cells	Fisher et al. (2011)
	IGF1		
	GRB2		
	KRS1		
miR-21	PTEN	Cells	Cheng and Zhang, (2010a)
	PDCD4		
	SPRY2		
miR-30	RUNX2	Cells	Zaragosi et al. (2011)
	RIP140		Hu et al. (2015)

Mapping common, antagonistic, and⁄or parallel regulatory targets in the health status of different organisms by epigenetic mechanisms is also highly dependent on biotechnology, bioinformatics, confirmatory approaches from the bench, and effective gain and loss of function protocols. Translational approaches from the bench to clinical confirmation are also crucial to show how mechanisms can interact or be changed in different complexity grades. We are moving towards a science where all the knowledge produced in these inter areas converge, thus generating increasingly accurate and individualized approaches for treatment, prevention, and detection of diseases that globally affect humanity.

## Glossary

ABCA1,: ATP-binding cassette transporter A
ADicerKO,: Knockout Dicer-deficient
AF,: Atrial Fibrillation
Akt,: Protein kinase B (PKB)
ALDH2,: Aldehyde dehydrogenase
AMPK,: AMP-activated protein kinase (α1 and α2 subunit)
ANF,: Atrial natriuretic factor
ApoE-/-,: Apolipoprotein E knockout mice
AT,: AT
BAT,: Brown AT
BCL6,: B-cell lymphoma 6
BeAT,: Beige AT
BIRC,: Baculoviral IAP Repeat Containing 6
BMI-,: Body mass index
BNP,: Brain natriuretic peptide
CAD,: Coronary vascular disease
CH-,: Cardiac hypertrophy
CNP,: Cardiac natriuretic peptides
CRP-,: C-Reactive Protein
CVD,: Cardiovascular disease
CVS,: cardiovascular system
CIDEA,: Cell Death Inducing DFFA Like Effector A
CRAT,: Carnitine O-acetyltransferase
EAT,: Epicardial AT; epicardial fat
EGR-1,: Early growth response protein 1
ERR-α,: Estrogen-related receptor α
EVs,: Extracellular vesicles
Fas,: Fas ligand protein
FGF21,: Fibroblast Growth Factor
FLT,: Follistatin
GDF-15,: Growth differentiation factor-15
GRB2,: Growth factor receptor-bound protein
HF,: Heart failure
Hif1α,: Hypoxia-inducible factor 1-alpha
hiPSC,: Human induced pluripotent stem cells
IGF1,: Insulin Growth Factor 1
IGFR1,: Insulin-like growth factor receptor
IHF,: Intrahepatic fat
IL-6,: Interleukin 6
IL-10,: Interleukin 10
IL-1β,: Interleukin-1 beta
KDM1A,: Lysine-specific histone demethylase 1A (LSD1)
KLF4,: Krüppel-like factor 4
KRS1: Kinase suppressor of ras 1
KO,: Knockout
LIFR,: Leukemia inhibitory factor receptor
LV,: left ventricle
LXR,: The liver X receptor
MAPK,: Mitogen-Activated Protein Kinases
MEG3,: Maternally Expressed Gene 3
MED13,: Mediator of RNA polymerase II transcription subunit 13
MHC,: myosin heavy chain
MII,: Myocardial infarct injury
miRNAs,: microRNAs
mRNA,: messenger RNA
mTOR,: Mammalian target of rapamycin
OBT,: Obesity
PAT,: Pericardial AT
PDCD4,: Programmed cell death 4
PGC1α,: Peroxisome proliferator-activated receptor gamma coactivator 1α
PI3Ks,: Phosphoinositide-3 kinases
PNUTS,: Protein phosphatase 1 binding protein
PPARγ,: Peroxisome proliferator-activated receptor gamma
PRDM16,: PR/SET Domain 16
PTEN,: Phosphatase and tensin homolog
PURβ,: Transcriptional activator protein Pur-beta
PVAT,: Perivascular AT; perivascular fat
RIP140,: Receptor-interacting protein 140
RNA,: ribonucleic acid
RUNX,: Runt-related transcription factor X
SEMA4b,: Semaphorin 4B
SIRT1,: Sirtuin 1
SMSC,: Skeletal muscle satellite cells
SMSC,: skeletal muscle satellite cells
SOX-6,: SRY-Box Transcription Factor 6
SPRY-2,: Sprouty homolog 2
SREBF1,: Sterol response element-binding factor-1
TGFβ,: Transforming growth factor beta
TGFβR1,: Transforming growth factor beta receptor
TNFα,: Tumor Necrosis Factor α
TRPV4,: Potential cation channel subfamily V member 4
TSC1,: Tuberous Sclerosis Complex 1
WAT,: White AT
UCP-1,: Uncoupling protein-1
UCP-3,: Uncoupling protein-3
3’-UTR,: 3’ untranslated region
VAT,: Visceral AT; visceral fat
VGFA,: Vascular endothelial growth factor A
VMP1,: Vacuole Membrane Protein 1
